# Top-Down Suppression of Sensory Cortex in an NMDAR Hypofunction Model of Psychosis

**DOI:** 10.1093/schbul/sby190

**Published:** 2019-04-03

**Authors:** Adam Ranson, Eluned Broom, Anna Powell, Fangli Chen, Guy Major, Jeremy Hall

**Affiliations:** 1 Neuroscience and Mental Health Research Institute, Cardiff University, Cardiff, UK; 2 School of Medicine, Cardiff University, Cardiff, UK; 3 Faculty of Medicine and Health Sciences, Universitat Internacional de Catalunya, Barcelona, Spain; 4 School of Biosciences, Cardiff University, Cardiff, UK; 5 School of Psychology, Cardiff University, Cardiff, UK

**Keywords:** psychosis, hallucination, schizophrenia, top-down, visual cortex, anterior cingulate cortex, NMDA receptor, MK-801

## Abstract

Conceptual and computational models have been advanced that propose that perceptual disturbances in psychosis, such as hallucinations, may arise due to a disruption in the balance between bottom-up (ie sensory) and top-down (ie from higher brain areas) information streams in sensory cortex. However, the neural activity underlying this hypothesized alteration remains largely unexplored. Pharmacological *N*-methyl-d-aspartate receptor (NMDAR) antagonism presents an attractive model to examine potential changes as it acutely recapitulates many of the symptoms of schizophrenia including hallucinations, and NMDAR hypofunction is strongly implicated in the pathogenesis of schizophrenia as evidenced by large-scale genetic studies. Here we use in vivo 2-photon imaging to measure frontal top-down signals from the anterior cingulate cortex (ACC) and their influence on activity of the primary visual cortex (V1) in mice during pharmacologically induced NMDAR hypofunction. We find that global NMDAR hypofunction causes a significant increase in activation of top-down ACC axons, and that surprisingly this is associated with an ACC-dependent net suppression of spontaneous activity in V1 as well as a reduction in V1 sensory-evoked activity. These findings are consistent with a model in which perceptual disturbances in psychosis are caused in part by aberrant top-down frontal cortex activity that suppresses the transmission of sensory signals through early sensory areas.

## Introduction

Schizophrenia is a neuropsychiatric disease characterized by psychotic symptoms including hallucinations (false perceptions); however the specific disturbances in neural activity that underpin these perceptual abnormalities are unclear. A number of computational theories based around predictive processing have been advanced, which propose that perceptual disturbances may arise due to a disruption in the balance between bottom-up (ie sensory) and top-down (ie from higher brain areas) information streams in sensory cortex. This imbalance could result in inappropriate or false inference because of an abnormal balance in perceptual processing between top-down signals and sensory evidence.^1–7^

According to the predictive coding framework, hierarchically higher brain regions send predictions to lower brain regions derived from an internal model of the environment. These predictions are compared to bottom-up input, and mismatches between the two generate ascending prediction errors that serve to improve the accuracy of the internal model. Physiologically, alterations in the influence of top-down predictive signals could result both from changes in activity in higher cortical regions providing descending signals and/or from alterations in local circuitry (such as shifts in excitatory/inhibitory balance) in lower brain regions in which comparisons between predictions and bottom-up signals are performed and prediction errors are encoded.^5–7^ At present, however, there remains little direct evidence of changes in psychosis of activity of neurons providing top-down signals to sensory cortex, or understanding of the influence this may have on sensory cortex activity.

Pharmacological *N*-methyl-d-aspartate receptor (NMDAR) antagonism presents a powerful experimental model in which to explore the neural activity alterations that might underlie perceptual disturbances in psychosis because of its acute effects and high face and construct validity.^8,9^ In humans administration of subanesthetic doses of NMDAR antagonists such as ketamine and phencyclidine produces symptoms of schizophrenia including disorganized thought and perceptual distortions,^10–12^ and exacerbate symptoms in drug-free schizophrenic patients.^13^ In addition, the influential dysconnection hypothesis proposes that aberrant functioning of other neurotransmitter systems, such as the dopamine system, could ultimately result in abnormal development of synapses mediated by NMDARs because of their modulatory influence on NMDARs during synaptic plasticity.^14^ Abnormal development of NMDARs may be particularly detrimental to circuitry mediating cortical feedback signals because of their role in mediating recurrent cortical inputs^14^ and in generating NMDA spikes in the tuft dendrites that receive long-range corticocortical input.^15,16^ Alteration in NMDAR function has also been strongly implicated in the pathogenesis of schizophrenia in large-scale genetic studies,^17–20^ including both directly through genes encoding NMDAR subunits and indirectly through modulators of NMDAR function.^14^

We used in vivo 2-photon calcium imaging in awake behaving mice to measure the effects of global NMDAR disruption on the activity of axons that provide dense top-down input to the primary visual cortex (V1). This projection is thought to mediate top-down attentional and predictive modulation of visual processing and targets both excitatory and several classes of inhibitory neurons in V1.^21–23^ Consistent with a shift in top-down/bottom-up balance, we find that global NMDAR hypofunction results in a large elevation in activity of ACC→V1 axons, and that surprisingly, this aberrant increase results in a net suppressive effect on V1 activity. These results are consistent with the notion of increased frontal top-down input to sensory cortex in psychosis and suggest that associated perceptual disturbances are generated in part through a net suppression of sensory cortex activity.

## Methods

### Animals

All experimental procedures were carried out in accordance with institutional animal welfare guidelines, and licensed by the UK Home Office. Experiments were carried out on adult mice (aged >P90), and no systematic randomization was used with respect to region labeled or other conditions. For experiments in which parvalbumin (PV) interneurons were labeled, this was achieved by crossing the B6.Cg-Gt(ROSA)26Sortm14(CAG-tdTomato)Hom/J and B6;129P2-Pvalbtm1(cre)Arbr/J (Jackson Laboratory, JAX Stock 007914 and 008069, respectively). Mice were housed under normal light conditions (14 h light, 10 h dark) and recordings were made during the light period.

### Animal Surgical Preparation and Virus Injection

Aseptic surgical procedures were conducted based on previously described protocols.^24,25^ Approximately 1 hour prior to cranial window surgery and virus injection, animals were administered with the antibiotic Baytril (5 mg/kg, s.c.) and the anti-inflammatory drugs Carprofen (5 mg/kg, s.c.) and Dexamethasone (0.15 mg/Kg, i.m.). Anesthesia was induced and maintained using isoflurane at concentrations of 4%, and 1.5%–2%, respectively. After animals were stereotaxically secured, the scalp and periosteum were removed from the dorsal surface of the skull, and a custom head plate was attached to the cranium using dental cement (Super-Bond C&B), with an aperture approximately centered over right V1, or retrosplenial cortex. For injections into V1 transcranial intrinsic signal imaging was then used to determine the precise location of V1,^26–28^ after which a 3-mm circular craniotomy was performed, centered on the area of V1 that responded to binocular visual stimulation. The V1 injections were targeted to the binocular primary visual cortex, defined as the region medial to the vertical meridian that was identifiable by the reversal of the direction of the horizontal progression of the retinotopic map. For injections into anterior cingulate cortex (ACC), a small craniotomy was first made over the region (0.3 mm lateral of bregma) either using a dental drill or by thinning the overlying skull and then piercing a small hole using a hypodermic needle, after which a larger 3-mm circular craniotomy was performed over V1. For injections into retrosplenial cortex for retrosplenial soma imaging, a 3-mm circular craniotomy was performed over the region (centered 0.4 mm lateral and 2.4 mm posterior to bregma). For imaging of retrosplenial cortex axons in V1, a small craniotomy was first made over retrosplenial cortex (centered 0.4 mm lateral and 2.4 mm posterior to bregma) either using a dental drill or by thinning the overlying and then piercing a small hole using a hypodermic needle, after which a 3-mm circular craniotomy was performed over V1. After craniotomy, injections of a virus to drive expression of GCaMP6s (AAV1.Syn.GCaMP6s.WPRE.SV40; titer after dilution 2×10^11^ GC/ml) were made into the relevant region (V1, layer 2/3 depth = 250 µm, layer 4 depth = 400 µm, 40 nl at 1–3 sites, anterior-posterior (AP) = −2.6 mm, lateral-medial (LM) = 2.8 mm; ACC, depth = 800 µm, 100 nl at 1 site, AP = 0.2, LM = 0.3; retrosplenial cortex for soma imaging, depth = 250 µm, 40 nl at 1–3 sites; retrosplenial cortex for axon imaging, 40 nl at 1 site, AP = −2.4 mm, LM = 0.5 mm; all coordinates measured relative to bregma). For ACC inactivation experiments, muscimol was injected into the ACC (coordinates as earlier) via a small craniotomy, with injections taking place under sterile conditions away from the 2-photon microscope (50 nl, 5 g/l). All injections were made using a microsyringe driver (WPI, UltraMicroPump) coupled to a pulled and beveled oil-filled glass micropipette with a tip outer diameter of approximately 30 μm. After injection the craniotomy was closed with a glass insert constructed from 3 layers of circular no. 1 thickness glass (1 × 5 mm, 2 × 3 mm diameter) bonded together with optical adhesive (Norland Products; catalog no. 7106). After surgery, animals were allowed at least 2 weeks to recover after which they were habituated to head fixation either during passive visual stimulation or during visual discrimination training.

### Imaging and Locomotor Behavior

In vivo 2-photon imaging was performed using a resonant scanning microscope (Thorlabs, B-Scope) with a 16 × 0.8 NA objective 3-mm working distance (Nikon). GCaMP6s and tdTomato were excited at 980 nm using a Ti:sapphire Laser (Coherent; Chameleon) with a maximum laser power at sample of 50 mW. Data were acquired at a frame rate of approximately 60 Hz and averaged, resulting in a frame rate of approximately 10 Hz. Cortical surface vascular landmarks were used to locate the same neurons between sessions. During 2-photon imaging, animals were free to run on a custom-designed fixed axis cylindrical treadmill, and movement was measured using a rotary encoder (Kübler, 05.2400.1122.0100). Imaging, behavioral, and visual stimulation timing data were acquired using Scanimage 4.1 and custom-written DAQ code (MATLAB) and a DAQ card (NI PCIe-6323; National Instruments). In vivo intrinsic signal imaging was performed using previously described methods^27^ using either a custom-built system based around a MAKO G-125B camera (AVT) or a commercially available system (Imager 3001; Optical Imaging Inc).

### Visual Stimuli

For V1 recordings, the preferred population retinotopic location of the field of view of neurons was determined in advance using circular 30° × 30° drifting horizontal square wave gratings with temporal frequency of 2 Hz and spatial frequency of 0.05 cycles per degree. Each stimulus appeared and was stationary for 5 seconds, drifted for 2 seconds, was stationary for 2 further seconds, and then disappeared. Trials were spaced by 3 seconds, during which a gray screen was displayed. Visual stimuli were generated in MATLAB using the psychophysics toolbox^29^ and displayed on calibrated LCD screens (Iiyama, BT481). Having established retinotopic preference, orientation tuning was next measured using circular gratings with the same temporal and spatial frequencies, at the identified preferred location, and displayed at 12 different orientations. For recordings of ACC axons in V1, retrosplenial cortex somata, or retrosplenial cortex axons in V1, the visual stimulus was positioned in the binocular area directly in front of the animal.

### Experimental Design

In order to measure the effect of NMDAR blockade, a 6-minute recording of baseline activity was made (either in V1 somata, ACC axons in V1, retrosplenial cortex somata or retrosplenial cortex axons in V1) during which the animal was exposed to horizontally oriented grating stimuli with the spatial and temporal frequencies and position determined as described earlier, which varied in size between 10° and 60° in steps of 10°. After this baseline period, the recording and visual stimulation were briefly paused; animals were injected with MK-801 (0.1 mg/kg, s.c.) or the same volume of saline, after which the recording and visual stimulation were resumed for 45 minutes. The dose of MK-801 was based on previous work in which it was shown to be effective in the rat prefrontal cortex (PFC) to induce increased excitability of pyramidal neurons and decreased excitability of interneurons.^10,30^ The experimenter was not blind to experimental group during the data collection or analysis.

### Calcium Imaging Data Analysis

Brain motion was first corrected for using an automated registration algorithm^31^ implemented in MATLAB, and data from the preinjection administration period were registered to the postinjection period. A 20 μm border was removed from all frames (more than the maximum brain movement observed) to ensure that all pixels were present in all frames in both sessions. Soma regions of interest (ROIs) were identified using a custom written semiautomated algorithm based on grouping of pixels with correlated time courses. Pixels within each region of interest were then averaged and background fluorescence contamination was estimated from a 30 μm circular area surrounding each soma ROI (excluding other ROIs) and subtracted from the soma ROI signal with a weighting of 0.7. Only cells with somata that were >5% brighter than the surrounding neuropil were included in further analysis. For analysis of axonal data, ROIs of the boutons were manually identified from the averaged movie frame. The time series of each ROI was then converted from a raw fluorescence value to ΔF/F with the denominator F value calculated as the median of the raw fluorescence trace during the baseline period. Population activity was calculated as the mean time course of all detected ROIs smoothed with a 2.5-minute sliding window. Correlation of excitatory and inhibitory populations was calculated by averaging the time courses of all identified excitatory and inhibitory neurons and then calculating the Pearson correlation coefficient during the baseline and post-saline/drug period. The time course of the coupling of excitatory and inhibitory populations was calculated by first smoothing the traces of both cell types with a sliding averaging window (window size of 5 s), then normalizing each trace to the baseline period (to control for baseline differences in levels of activity in the excitatory/inhibitory populations), and then dividing the excitatory trace by the inhibitory trace at each time point. To identify tdTomato-labeled PV+ neurons, cells were semiautomatically classified based on a thresholded mean registered red channel image, which was eroded and then dilated to remove small areas of labeling of neural processes, after which classification was verified manually.

### Analysis of Visually Evoked Activity

For analysis of visually evoked activity tuning curves, the average responses of neurons to each visual stimulus were calculated, and the maximum ΔF/F value during the 2-second drifting phase was taken as the neuron’s response amplitude. Visual stimulus trials in which animals were moving were excluded. Size tuning data were fit using a difference-of-Gaussians model^32^

$$\begin{array}{l} R\left(s\right)=R_{b}+K_{e}\int_{-s/2}^{s/2}e^{-{\left(2y/a\right)}^{2}}dy-K_{i}\int_{-s/2}^{s/2}e^{-{\left(2y/b\right)}^{2}}dy \end{array}$$

Here, *R*(*s*) is the response to size *s*, *R*_*b*_ is the baseline response, and the 2 integrals represent excitatory and inhibitory components. *K*_*e*_ and *K*_*i*_ are the gain of the excitatory and inhibitory components, and *a* and *b* represent space constants. Surround suppression index (SSI) was defined as (R_pref_−R_max_)/R_pref_ where R_pref_ is the response to the preferred fitted size and R_max_ is the fitted response to the largest size of stimulus. Parameters were fit using the EzyFit MATLAB toolbox by a nonlinear minimization of the sum of squared residuals.

### Statistical Methods

Statistical analysis was carried out in MATLAB 2016b using the Statistics toolbox, and group average values are presented throughout as mean ± standard error of the mean or median with error bars indicating interquartile range unless otherwise noted. The statistical significance of comparisons between groups was determined using a 2-sided *t* test or ANOVA, or Kruskal–Wallis test unless otherwise noted, and *P* values < .05 were considered significant (*P < .05, **P < .01, and ***P < .001). Similarity of variance and normal distribution was checked with the vartestn MATLAB function. Correction of *P* values for multiple comparisons was calculated using the MATLAB function multcompare using the Tukey–Kramer method. Approximate group sizes were based on typical sizes used in this field in similar experiments.

### Histology

Mice were deeply anesthetized with sodium pentobarbital (60 mg/kg, i.p.; Euthatal; Merial Animal Health) and transcardially perfused with 0.1 M phosphate-buffered saline (PBS) followed by 4% paraformaldehyde (PFA) in 0.1 M PBS. The brains were removed and placed in PFA for >4 hours before being transferred to 25% sucrose overnight at room temperature, with gentle agitation. A 1-in-3 series of coronal sections (40 µm) was cut on a freezing microtome and mounted immediately on gelatin-coated slides. Sections were left to dry overnight before being mounted and coverslipped using VECTASHIELD Antifade mounting medium (Vector Laboratories Ltd). Mounted sections were imaged on a Leica DM5000B microscope using a Leica DFC310FX digital camera and Leica Application Suite image acquisition software.

## Results

**Fig. 1.**
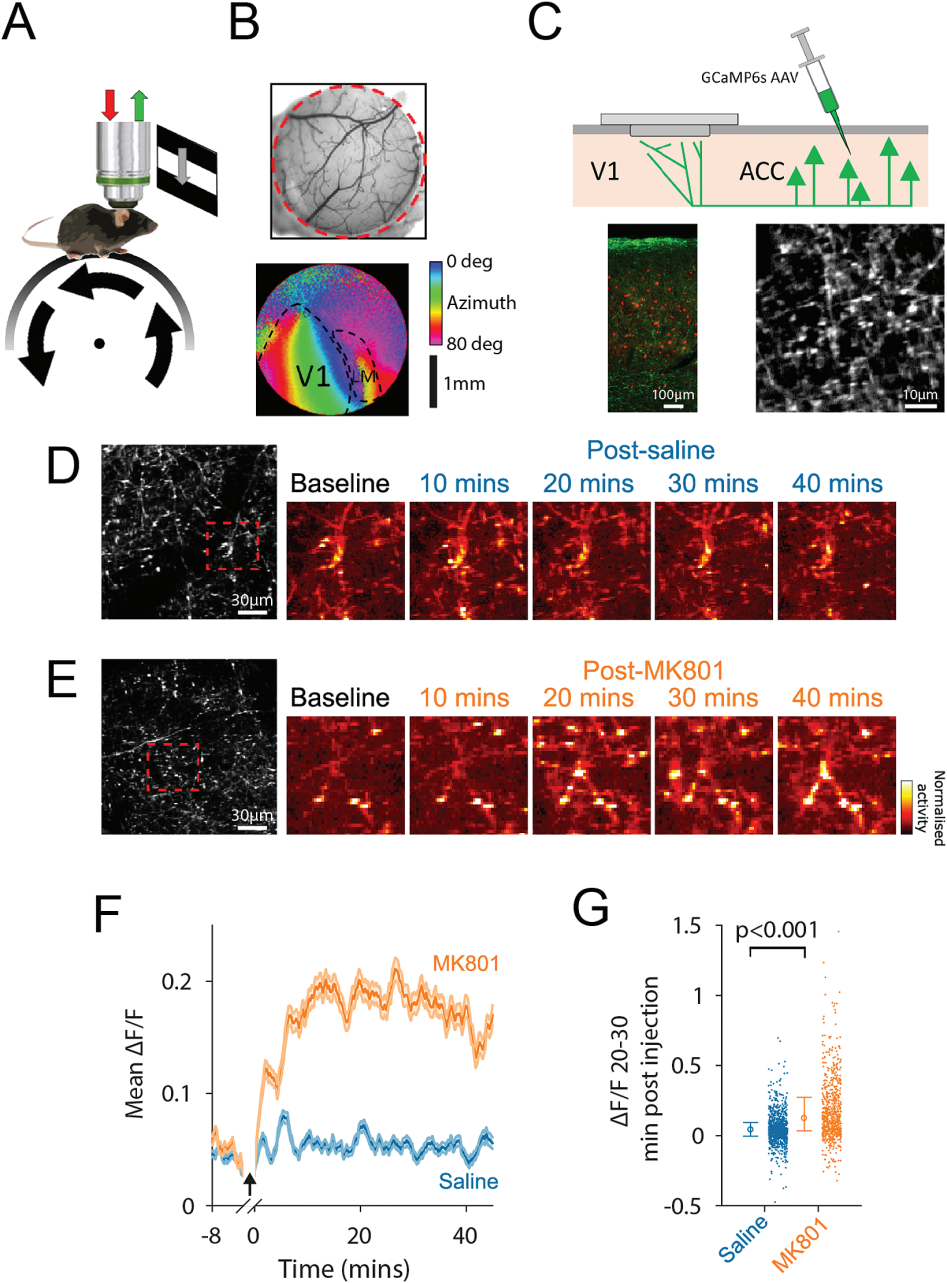
Increased activation of ACC→V1 axons during global *N*-methyl-d-aspartate receptor (NMDAR) block. (**A**) Schematic of visual stimulation and recording setup. (**B**) Cranial window and intrinsic signal retinotopic map of V1 and higher visual area LM. (**C**) Schematic of anterior cingulate cortex (ACC) axon recording configuration, and ex vivo (side view) and in vivo images of recorded tissue (red indicates parvalbumin (PV) + interneurons). (**D–E**) Example activity maps of ACC→V1 axons at baseline and after administration of saline (D) or MK-801 (E). (**F**) ACC→V1 axon activity after saline (blue) or NMDAR block (orange). (**G**) Average activity of ACC→V1 axons at 20–30 minutes post injection during visual stimulation. In figures showing averaged ACC axon data *n*_(saline)_ boutons = 935 from six mice, *n*_(MK-801)_ = 728 boutons from seven mice.

To investigate the effects of global NMDAR hypofunction on top-down ACC→V1 signals, we used in vivo calcium imaging in awake mice to record from ACC axon terminals in V1 (figure 1A). Recordings were targeted to V1 using intrinsic signal functional imaging (figure 1B). Activity of top-down projections to V1 from ACC was recorded by transfecting ACC neurons with GCaMP6s and recording GCaMP6s-filled axons at their termination site in layer 1 of V1 during visual stimulation (figure 1C). After a baseline recording and visual stimulation period, animals were administered with either a subanesthetic dose of the NMDAR antagonist MK-801^10,30^ (NMDAR block: 0.1 mg/kg s.c.) or the same volume of saline, and recording was then continued for 45 minutes under the same stimulation conditions. The dose of MK-801 was chosen based on previous work in rat PFC, which found it to be effective in inducing behavioral changes and modifying excitatory/inhibitory balance.^10,30^ Analysis was limited to nonrunning periods to control for behavioral changes associated with NMDAR block (Mohn et al.^33^ and Supplementary Figure S1-1A). Under saline control conditions, we found that ACC axons exhibited no significant change in activity over the course of the experiment. In contrast, following NMDAR block, we found that ACC→V1 axons exhibited an approximate 3-fold increase in activity (Kruskal–Wallis test: *P* < 10^–39^; figures 1D–G; see Supplementary Figure S1-1B for analysis of moving periods).

**Fig. 2.**
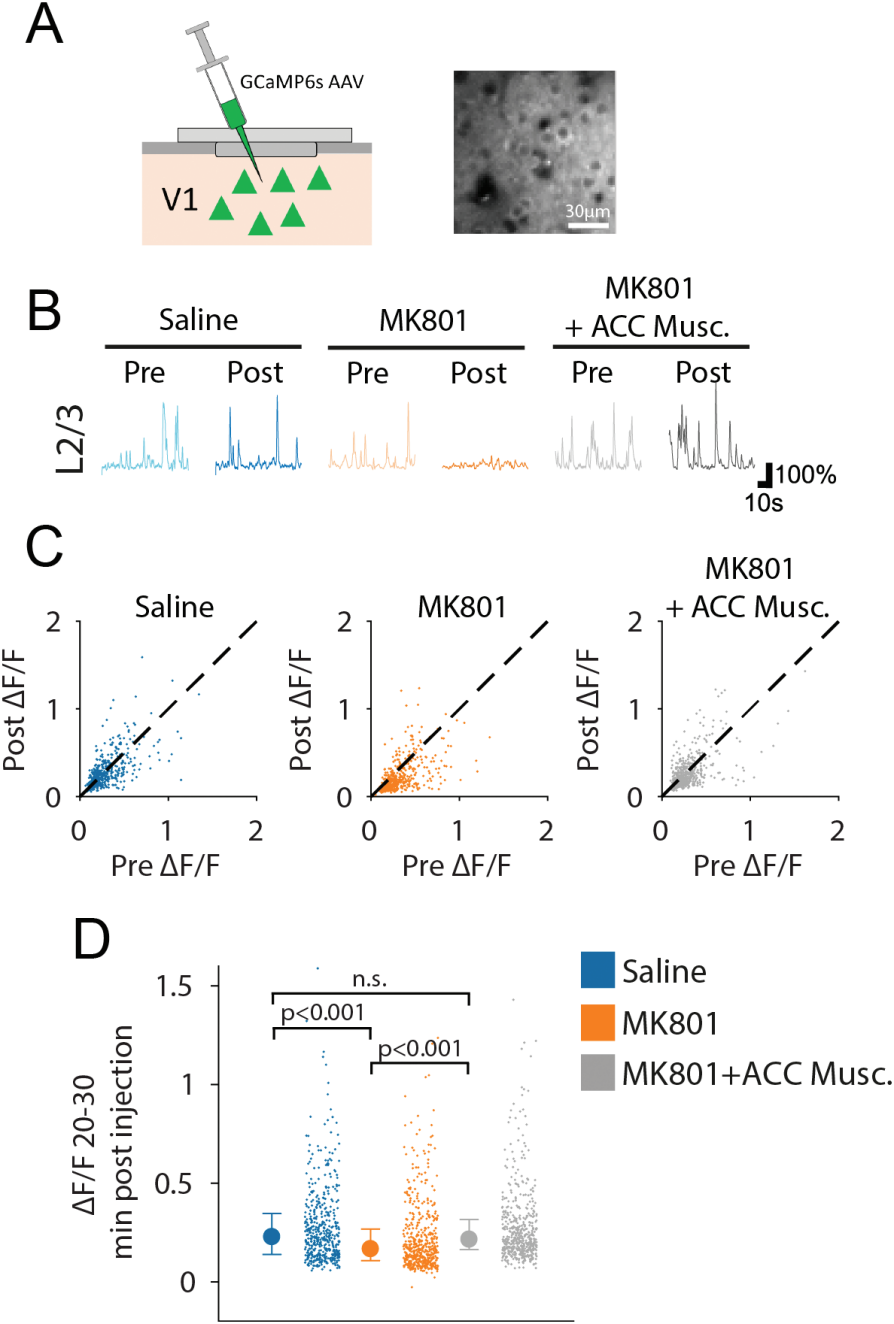
Anterior cingulate cortex (ACC)-dependent suppression of layer 2/3 spontaneous activity by *N*-methyl-d-aspartate receptor (NMDAR) block. (**A**) Schematic of V1 labeling (left) and example V1 field of view of labeled neurons (right). (**B**) Example traces of layer 2/3 neuron spontaneous (dark) activity in each treatment condition. (**C**) Comparison of mean neural activity before and after each drug treatment condition. (**D**) Average neural activity during stationary periods, in layer 2/3 neurons following saline (blue), NMDAR block (orange), or NMDAR block + ACC muscimol inactivation (gray). *n*_(saline)_ = 6 mice, 753 layer 2/3 neurons, *n*_(MK-801)_ = 7 mice, 704 layer 2/3 neurons, *n*_(MK-801+Musc)_ = 5 mice, 530 layer 2/3 neurons.

Axons projecting from ACC to V1 are known to target both excitatory and subclasses of inhibitory neurons in V1, and could consequently have an overall net excitatory or inhibitory effect on V1 activity.^21,34^ We therefore next asked what influence NMDAR block-driven increased ACC top-down input might have on V1 activity. To address this question, we measured spontaneous activity in layer 2/3 V1 neurons, labeled with an AAV-driving expression of GCaMP6s (figure 2A). Recordings were made in darkness, during either NMDAR block or saline treatment, and analysis was limited to stationary periods. In contrast to our observations of ACC axons, and in vivo reports from PFC,^10,30^ we found that NMDAR block reduced spontaneous activity in V1 by approximately 35%, whereas no significant effect was observed after saline treatment (Kruskal–Wallis test: *P* < 10^–9^; figures 2B and C; see Supplementary Figure S1-1B for analysis of moving periods). We tested for possible dependence of this reduced activity of layer 2/3 neurons on the previously observed elevated ACC activity by acutely inactivating the ACC through local muscimol injection (50 nl, 5 g/l) during global NMDAR block. ACC inactivation significantly reduced suppression of layer 2/3 activity by NMDAR block to approximately 14% (Kruskal–Wallis test: *P* < 10^–9^; figures 2B–D), indicating a causal role for ACC input in mediating global NMDAR block-induced V1 suppression. The differences described between V1 (reduced activity) and ACC (increased activity) could be due to differences in the recorded neuronal compartment, or differential effects of MK-801 on axons vs somata. To explore this possibility, we made calcium imaging recordings from the retrosplenial cortex (Supplementary Figure S1-1B and C), which also provides top-down input to V1 (Makino et al.^35^Supplementary Figure S1-1C right panels), and in which both axons and somata were optically accessible in vivo, which was not the case for the ACC. These data indicate that the signals recorded in somata and axons are comparable, and additionally that the retrosplenial cortex exhibits a similar reduction in activity with NMDAR block as V1 (Supplementary Figure S1-1).

**Fig. 3.**
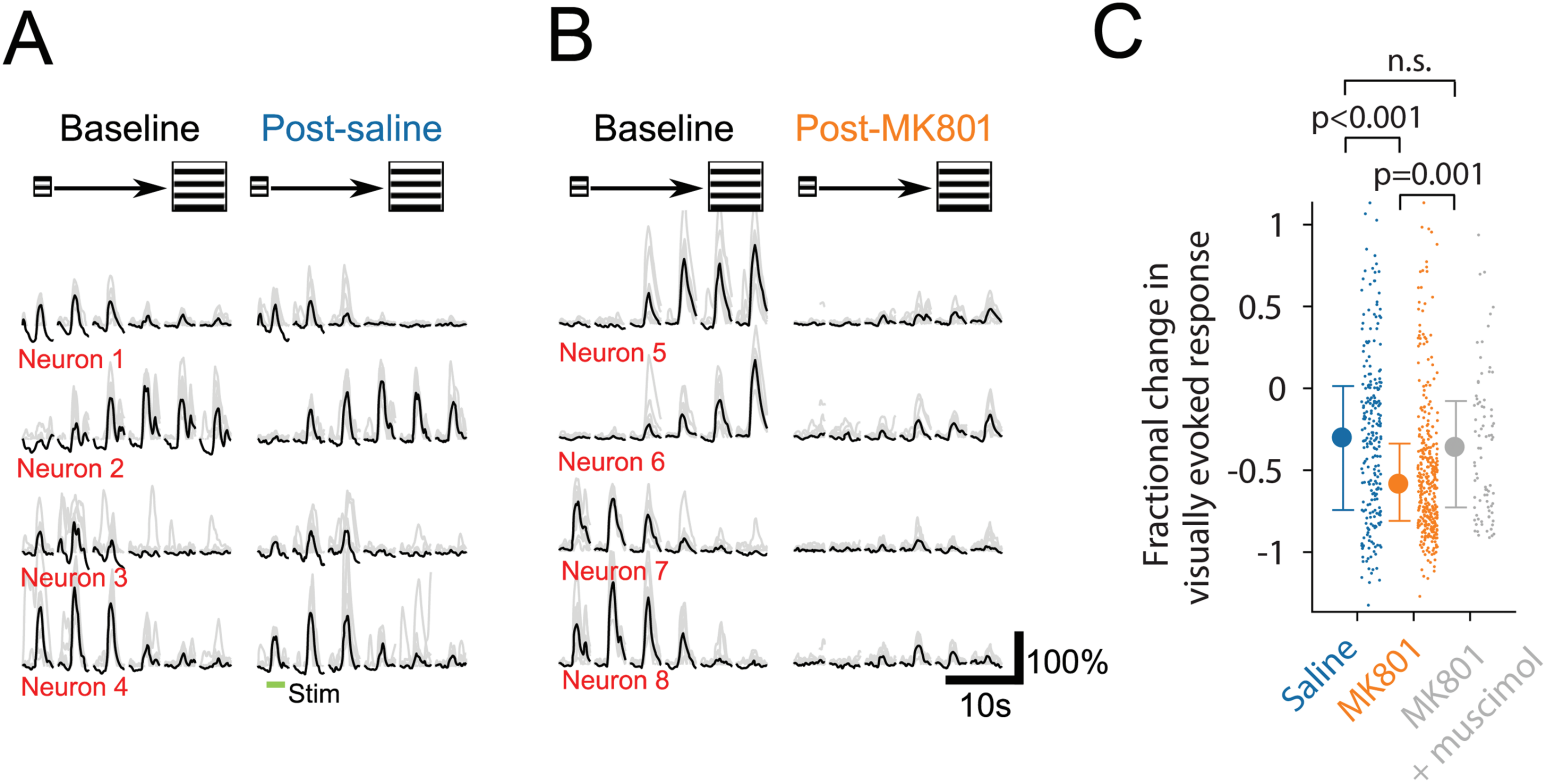
Visually evoked activity of layer 2/3 neurons is suppressed by global *N*-methyl-d-aspartate receptor (NMDAR) block. (**A**–**B**) Examples traces of individual sweeps (gray) and averaged (black) grating stimulus-evoked responses of single V1 neurons pre- and postinjection of saline (A) and MK-801 (B). Range of stimulus sizes is schematically illustrated above traces. (**C**) Fractional change in visually evoked response to preferred stimulus of individual neurons at 20−30 minutes postinjection. *n*_(saline)_ = 7 mice, 250 visually responsive neurons; *n*_(MK-801)_ = 9 mice, 369 visually responsive neurons; *n*_(MK-801 + musc)_ = 7 mice, 84 visually responsive neurons.

We next tested if sensory-evoked activity in V1 was attenuated in a similar manner to spontaneous activity during global NMDAR block. Consistent with our observations of V1 spontaneous activity, NMDAR block also reduced the visually evoked response of V1 layer 2/3 neurons, resulting in a fractional reduction in response to preferred stimulus of 60% which compares to the 31% reduction observed under control conditions, presumably due to adaptation with repeated stimulus presentation (Kruskal–Wallis test: *P* < 10^−5^; figures 3A–C). Inactivating ACC through local muscimol injection (50 nl, 5 g/l) during global NMDAR block resulted in a partial recovery of visually evoked activity to 37% below baseline (relative to MK-801 condition; Kruskal–Wallis test: *P* = .01; figure 3C), again suggesting a causal role for the ACC in suppression of sensory cortex activity. Previous reports have provided evidence for a differential effect of NMDAR block on putative excitatory neurons (increased activity) vs fast-spiking-type inhibitory neurons (decreased activity) in PFC in the rat.^10,30^ To examine the effect of NMDAR block on similar subpopulations in V1 in the mouse, we crossed a PVcre mouse line with a tdTomato reporter line, which was then injected with an AAV expressing GCaMP6s (Supplementary Figure S2-1A). This allowed us to record from PV+ interneurons as well as the remainder of the population that are expected to be almost entirely pyramidal neurons (putative pyramidal population). We found that the activity of the 2 populations was highly correlated under control conditions (mean R = 0.81 ± 0.04, *n* = 7 mice; Supplementary Figure S2-1B and C). We then compared the activity of the 2 populations during NMDAR block and found an approximate 40% suppression of both (2-way ANOVA, *P* = .0002; Supplementary Figure S2-1D and F) with no significant statistical interaction with cell type (*P* = .95). When compared to our observations of ACC axons (presumed to belong to excitatory neurons) and previous results in frontal cortex,^10,30^ these findings suggest significant interareal differences in the effect of global NMDAR block on excitatory neuron-mediated circuit output.

**Fig. 4.**
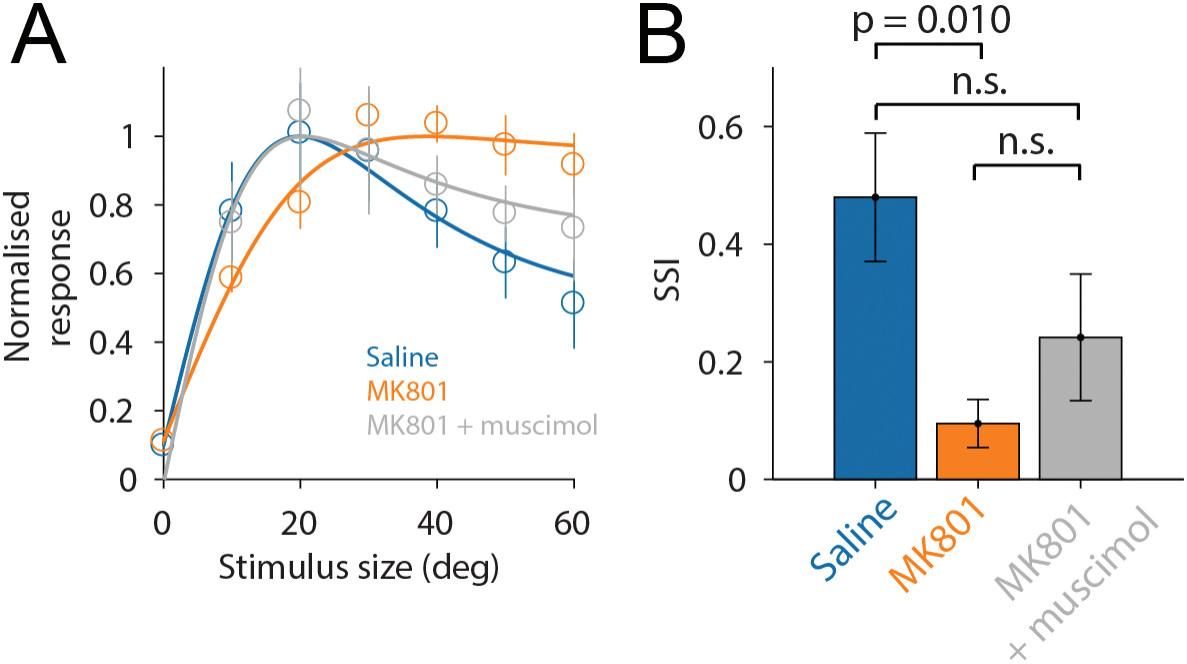
*N*-methyl-d-aspartate receptor (NMDAR) block alters surround suppression of V1 neurons. (**A**) Average population size tuning following saline or MK-801 administration, or MK-801 administration with anterior cingulate cortex (ACC) muscimol injection. (**B**) Average surround suppression index (SSI) in each condition. All data are presented as mean ± standard error of the mean or median, and numbers of animals and neurons are as in figure 3.

Previous studies in humans have reported evidence of weakened visual surround suppression in schizophrenic subjects.^36,37^ Surround suppression is known to vary with stimulus contrast which in turn modulates visual response amplitude, and so we next investigated whether the observed suppression of V1 visually evoked activity might mimic the effects of reduced stimulus contrast and result in reduced surround suppression. To test if NMDAR block resulted in any shift in population surround suppression of V1 neurons, we calculated average pre- and posttreatment responses of V1 neurons to each stimulus and then fit a difference-of-Gaussians model to the mean population response.^32^ To simplify interpretation, analysis was limited to stationary periods as locomotion is known to modulate surround suppression.^38,39^ An SSI was then calculated from the fitted data using the formula (R_pref_ − R_max_)/R_pref_ where R_pref_ is the response to the preferred size and R_max_ is the response to the largest size of stimulus. Consistent with previous reports in schizophrenic subjects, we found that following NMDAR block, population surround suppression was significantly less than with saline treatment (mean SSI saline 0.48 ± 0.11, and MK-801 0.09 ± 0.04, 1-way ANOVA, *P* = .010; figures 4A and B). There was no statistically significant change in SSI following ACC inactivation (1-way ANOVA, P = .43).

## Discussion

In summary, consistent with the idea of increased frontal top-down signals to sensory cortex underlying psychotic perceptual symptoms, during NMDAR hypofunction we observed a sustained increase in activity of axons projecting from the ACC to V1. This elevation in ACC top-down was associated with an ACC-dependent net suppression of spontaneous V1 activity and a reduction in visually evoked activity in V1. Reductions in population visual surround suppression were also observed with NMDAR block, which appeared at least in part to be independent of ACC activity.

Perceptual disturbances in psychosis, such as hallucinations, could conceivably come about through either a loss or degradation of visual information relay, or an increase in influence of internally generated signals carrying information such as predictions. Our results suggest the perhaps counterintuitive possibility that perceptual disturbances in psychosis do come about through a reduction in the amplitude of visual signals being relayed through the primary visual cortex, but that this is driven by increased activity of neurons providing top-down internally generated signals. If the activity of some neurons in sensory cortex signals prediction error or a mismatch from expectations as suggested by predictive coding theories and experimental evidence,^40,41^ then the aberrant top-down suppression of these signals may result in a failure to update the internal representation or model of the world and a degradation of the ensuing predictions. Further disorder in predictive coding may come about from an inappropriate precision weighting of prediction errors^5,7^ due to both disruption of postsynaptic weights in the neurons that implement prediction error generation and unusually strong and possibly high noise synaptic input from ACC neurons providing predictions.

Physiologically, the suppressive effects observed in V1 neurons could come about in a number of ways. One possibility is that suppression is mediated through specific subclasses of inhibitory neurons within V1, such as SOM or VIP interneuron, which are known to be targeted by ACC axons.^21^ Consistent with this notion, SOM neurons are also known to play a role in mediating surround suppression^42^ and have been proposed to form an important component of circuitry in visual cortex responsible for generating prediction errors^43^ and mismatch negativity signals.^44^ Another possibility is that the effects observed are mediated through an intermediate brain region such as the thalamic reticular nucleus, which may in turn suppress feedforward thalamic visual input to V1.^22,45^ It is likely that both of these mechanisms contribute to the alterations in sensory processing observed. In future work, it will be important to further distinguish between these possibilities by probing the activity of other interneuron subtypes in this model. It is also important to note that NMDARs contribute to direct excitatory top-down cortical input to pyramidal neurons.^14–16^ In addition to interneuron-mediated suppressive effects of top-down input on excitatory neurons, it is expected that NMDAR block will also lead to a reduction in direct top-down excitatory input to pyramidal neurons.

Another outstanding question, not addressed by this study, is why the ACC exhibits an increase in activity of excitatory neurons, whereas in V1 and retrosplenial cortex we observe a decrease in activity, both of putative excitatory and PV+ inhibitory neurons. Previous work in the prefrontal cortex has also reported increased activity of regular spiking neurons and linked this to decreased activity of fast-spiking GABAergic interneurons.^10,30^ Similar regional variations have been reported in the neurotoxic effects of systemically administered MK-801 and other NMDAR antagonists such as Phencyclidine.^46^ These apparent regional differences may be due to differences in the sources of input received by different cortical areas or potential local differences in the regions in question in local connectivity patterns, expression of levels of NMDARs in difference cell types, or complement of different classes of interneuron.

Given the convergence of genetic and immunological risk factors for schizophrenia and related disorders on the NMDA receptor complex and associated synaptic components,^20^ these findings may be broadly relevant to the pathogenesis of altered cortical processing across a range of psychiatric disorders and cortical areas.

## Funding

This work was supported by a Wellcome Trust Strategic Award (100202/z/12/z) to Michael J. Owen, J.H., Lawrence Wilkinson, Adrian Harwood, Meng Li, David Linden, John Aggleton, Vincenzo Crunelli, and Derek Jones, a Wellcome Trust ISSF Seedcorn Award (105613/Z/14/Z) to A.R. and a Sêr Cymru Fellowship (80762-CU-080) to A.R.

## Supplementary Material

sby190_suppl_Supplementary_Figure_1

sby190_suppl_Supplementary_Figure_2
