# Supplementary figures and images for: Top-Down Suppression of Sensory Cortex in an NMDAR Hypofunction Model of Psychosis
Source: Schizophr Bull. 2019 Apr 3;45(6):1349–57. doi: 10.1093/schbul/sby190 (PMC6811829; doi:10.1093/schbul/sby190)

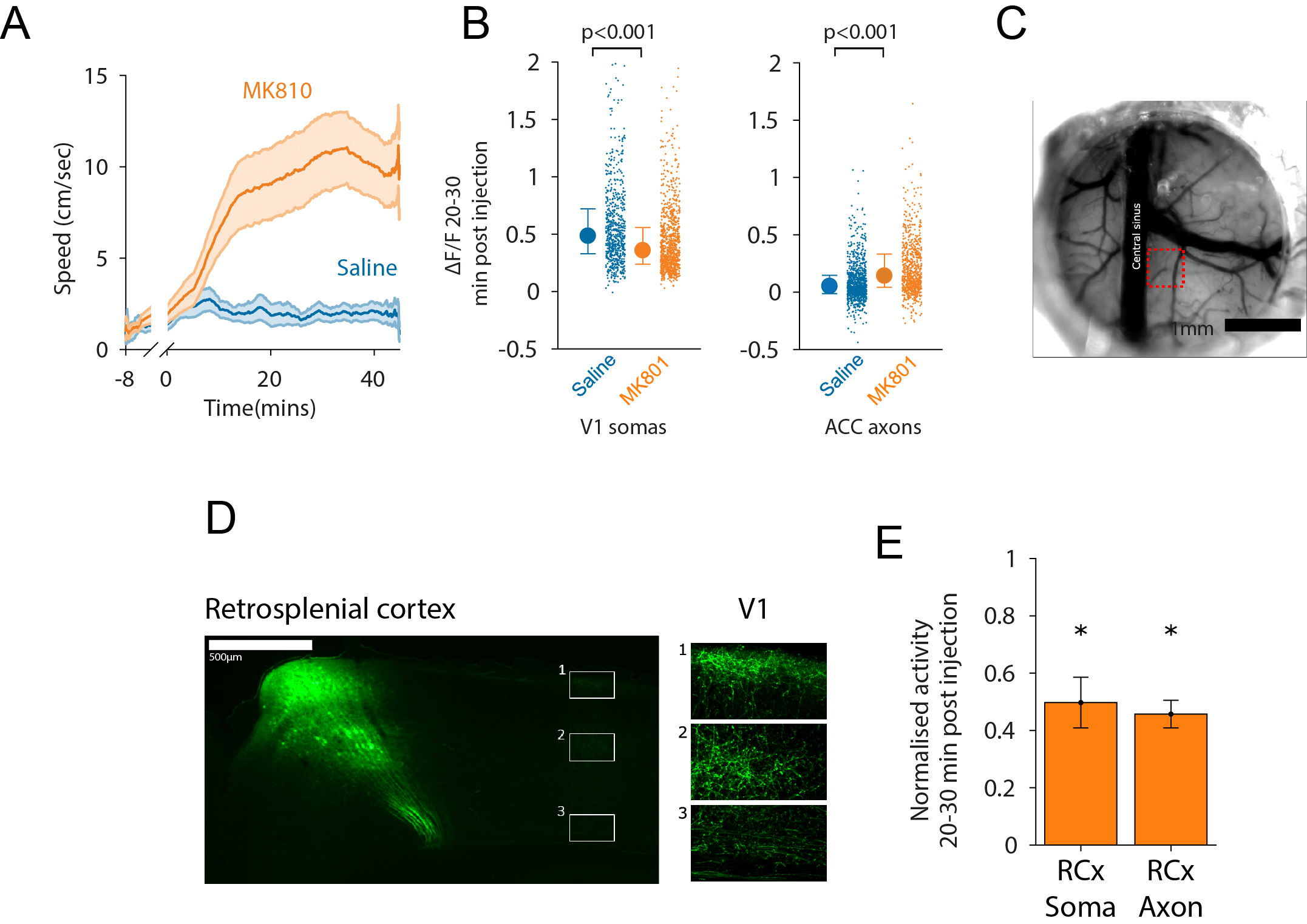

Supplement: sby190_suppl_Supplementary_Figure_1 [file sby190_suppl_supplementary_figure_1.png]

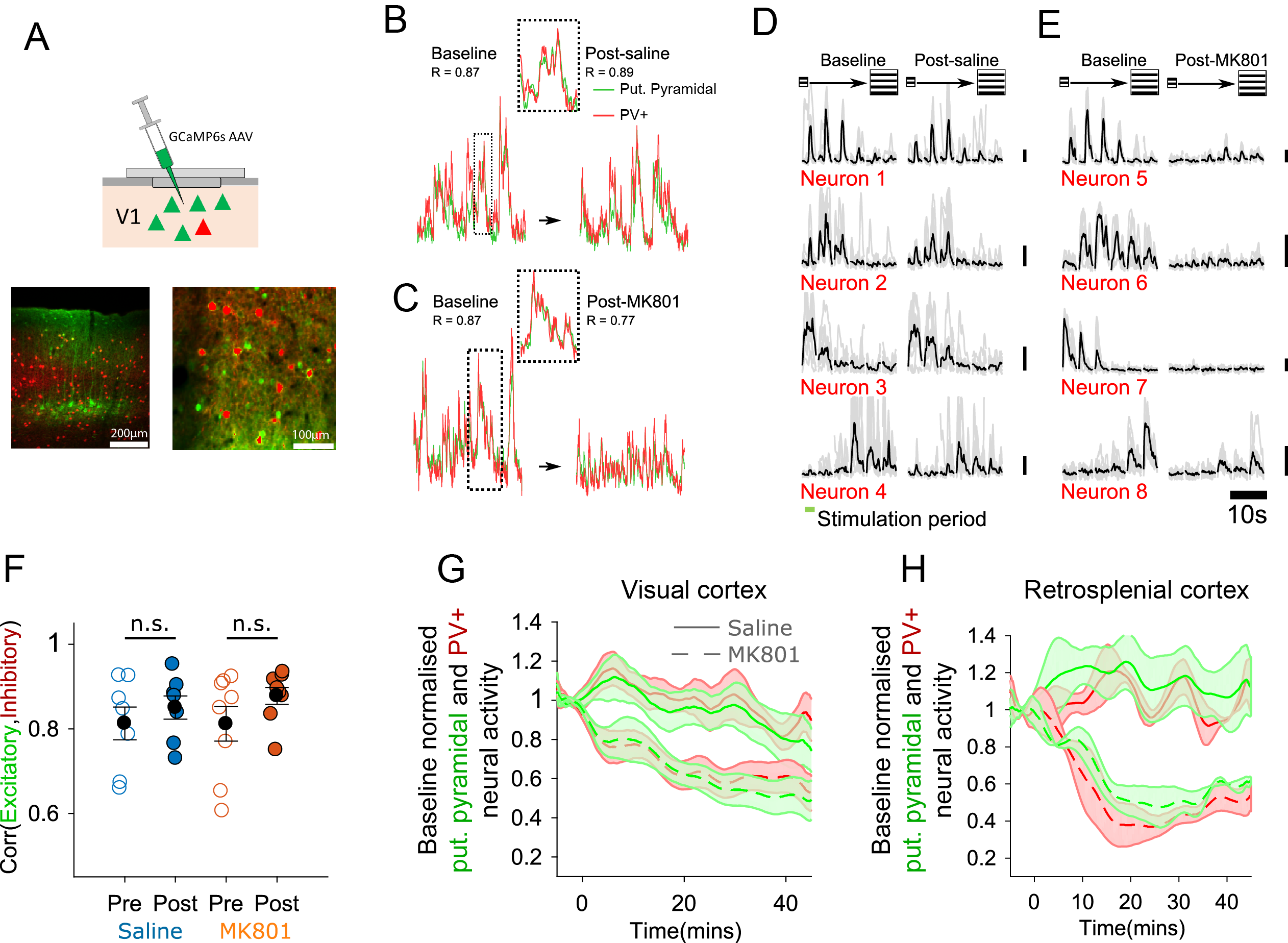

Supplement: sby190_suppl_Supplementary_Figure_2 [file sby190_suppl_supplementary_figure_2.png]
